# Charge Transfer Tuned by the Surrounding Dielectrics in TiO_2_-Ag Composite Arrays

**DOI:** 10.3390/nano8121019

**Published:** 2018-12-07

**Authors:** Yaxin Wang, Chao Yan, Chunxiang Li, Ziyang Lu, Changchang Ma, Yongsheng Yan, Yongjun Zhang

**Affiliations:** 1 School of Chemistry and Chemical Engineering, Jiangsu University, Zhenjiang 212013, China; wangyaxin1010@126.com (Y.W.); luziyang126@126.com (Z.L.); machang719@163.com (C.M.); yys@ujs.edu.cn (Y.Y.); 2 Zhonggong Education and Technology Co., Ltd., Changchun 130000, China; 18686656043@163.com; 3 College of Materials and Environmental Engineering, Hangzhou Dianzi University, Hangzhou 310018, China

**Keywords:** TiO_2_-Ag composites, electronic transfer, surrounding dielectrics

## Abstract

TiO_2_/Ag bilayer films sputtered onto a 2D polystyrene (PS) bead array in a magnetron sputtering system were found to form a nanocap-shaped nanostructure composed of a TiO_2_-Ag composite on each PS bead, in which the Ag nanoparticles were trapped partially or fully in the TiO_2_ matrix, depending on the TiO_2_ thickness. X-ray Photoelectron Spectroscopy (XPS) results showed the opposite shifts of binding energy for Ti 2p and Ag 3d, indicating the transfer of electrons from metallic Ag to TiO_2_ owing to the Ag-O-TiO_2_ composite formation. UV-Vis absorption spectra showed the blue shifts of the surface plasma resonance peaks, and the maximum absorption peak intensity was obtained for TiO_2_ at 30 nm. The surface-enhanced Raman scattering (SERS) peak intensity first increased and then decreased when the TiO_2_ thickness changed. The observations of SERS, XPS, and UV-Vis absorption spectra were explained by the dependency of the charge-transfer process on TiO_2_ thickness, which was ascribed to the changing dielectric properties in the metal/semiconductor system.

## 1. Introduction

When excited by a light incident on a metal surface, free electrons show a collective oscillation, known as surface plasma [1]. Surface plasma in nanostructures leads to an enhanced local electromagnetic field, which has excellent applications in many fields such as biomolecule analysis, pollution material degradation, energy conversion, and surface-enhanced Raman scattering (SERS) [2,3,4,5,6]. In these studies, the noble metals Au and Ag have been widely investigated due to their unique plasma characteristics, however, their applications are largely limited by shortcomings such as high cost, poor stability, and no reusability. To overcome these shortcomings, photocatalytic self-cleaning materials have recently been developed by combining plasmonic metal with conventional semiconductor photocatalysts such as TiO_2_ [7,8,9,10] and ZnO [11], which makes the materials recyclable to reduce the potential cost. TiO_2_ is an excellent photocatalyst material due to its good physical and chemical stability and high photocatalytic activity [12,13,14]. However, the quantum efficiency of TiO_2_ is greatly limited due to its quick recombination of photogenerated electrons and holes [15]. One main approach to improving the efficiency of such materials is the addition of nanoparticles to the titanium surface, which can trap electrons and lessen the recombination of electron-hole pairs [16,17]. TiO_2_ modified with Ag has been proven to restrain the recombination of photo-excited electrons and holes, which can improve the photocatalytic performance [18]. In our recent work, nanocap arrays of TiO_2_/Ag and co-sputtering TiO_2_-Ag were fabricated on two-dimensional colloidal arrays [19]. A significant SERS enhancement was observed when the sublayer Ag was 10 nm compared to the pure Ag monolayer, which is mainly ascribed to the charge–transfer effect.

Since the charge–transfer process depends on the surrounding dielectric properties, the charge–transfer behavior can be tailored by the materials around the metals. In this paper, we prepare a composite TiO_2_-Ag array on 2D polystyrene (PS) colloidal spheres. TEM measurements show Ag nanoparticles embedded in the semiconductor TiO_2_ matrix. When the TiO_2_ thickness changes, the morphology of each unit changes from partially-trapped Ag nanoparticles to fully-trapped Ag nanoparticles in the TiO_2_ matrix. XPS, SERS, and UV-Vis absorption spectra measurements indicated the charge–transfer process in our TiO_2_-Ag composite, which was attributed to the change in the surrounding dielectrics.

## 2. Experimental Section

### 2.1. Materials

The probing molecules are 4-Aminothiophenol (PATP) and Methylene blue (MB), with a purity of 99.9%. Sodium dodecyl sulfate and ethanol were purchased from Sigma Aldrich (St., Louis, MO, USA). The polystyrene (PS) colloidal beads were purchased from The Duke Co., Ltd., (Palo Alto, CA, USA) with a concentration of 10 wt % and a particle deviation less than 10%. Ag and TiO_2_ targets were supplied by Beijing TIANRY Science and Technology Developing Center (Beijing, China), with a purity 99.99% (wt %). Silicon wafers were supplied by Hefei Kejing Materials Technology Co., Ltd. (Hefei, China).

### 2.2. Preparation and Characterization

The film was deposited in a magnetron sputtering system model JGP-560C (Shenyang, China), with a base vacuum of 2 × 10^−4^ Pa and an argon pressure of 0.6 Pa. The sputtering power 50 W was applied to the target TiO_2_ and the sputtering power 21.6 W was applied to the target Ag. A PS (200 nm) monolayer was assembled on a modified Si substrate. First, the PS colloidal solution and ethanol were mixed with the same volume ultrasonically. The Si substrate covered with the mixture was submerged in water. The PS monolayer formed on the water surface, which was picked up by a new Si substrate. The ion beam etch technique was used to separate the PS beads from each other, using 2000 eV of energy and an etching time of 300 s. To realize saturation adsorption, the samples were immersed in PATP (3%) for more than 30 min. The morphology and microstructure measurements were performed by field emission scanning electron microscopy (FESEM) under an accelerating voltage of 5.0 KV and transmission electron microscopy (TEM) on JEM-2100HR (JEOL, Tokyo, Japan). UV-Vis spectra were obtained on a spectrophotometer, model Shimadzu UV-3600 (Kyoto, Japan). X-ray photoelectron spectroscopy was carried out by the Thermo Fisher Scientific (Waltham, MA, USA) system to determine the elemental composition and chemical state. Raman spectra were obtained with a Renishaw Raman (London, UK) system model 2000 confocal microscopy spectrometer with a spectral resolution of 1 cm^−1^. An air-cooled argon ion laser with 514.5 nm radiation (40 mW, power out of 1%) was used for the SERS. The spectra were recorded with an accumulation time of 10 s. 

## 3. Results and Discussion

**Figure 1:**
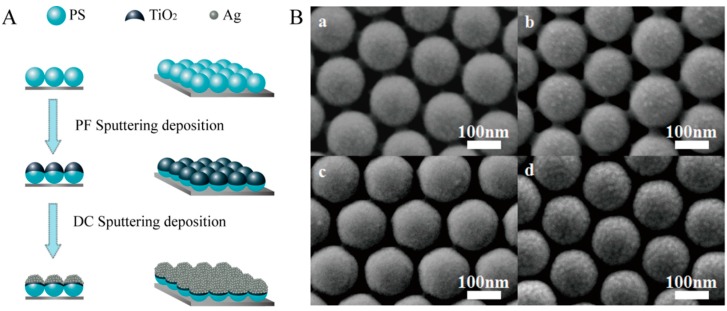
Schematic diagram of (**A**) the preparation process; and (**B**) field emission scanning electron microscopy (FESEM) images of the nanocap arrays for the TiO_2_ (t nm)/Ag (10 nm) bilayer (t = 10 nm, 20 nm, 30 nm, 40 nm from **a**–**d**).

Figure 1 shows the schematic for fabrication as well as the FESEM images of the nanocaps TiO_2_ (t nm)/Ag 10 nm (t = 10 nm, 20 nm, 30 nm, 40 nm). The PS beads were isolated from each other. When the TiO_2_/Ag film was deposited onto the PS beads, the isolated TiO_2_/Ag cap formed on each PS bead without connection with the neighbors, as the film thickness was far smaller than the bead radius. The aggregations of Ag particles become obvious and the surface roughness increased as the TiO_2_ thickness increased from 10 nm to 40 nm.

**Figure 2:**
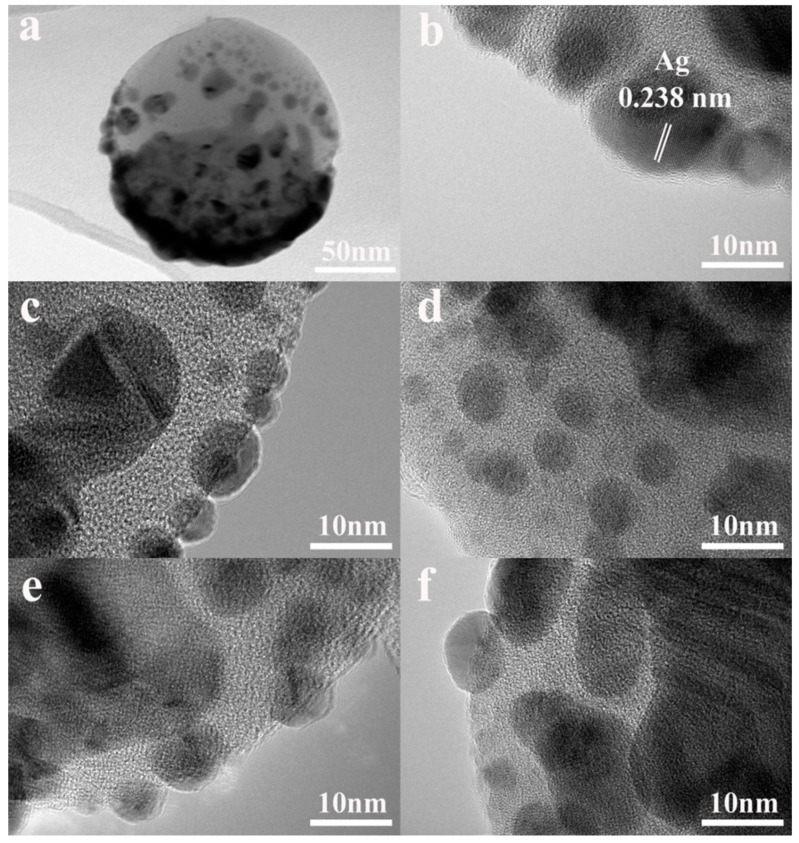
TEM and HRTEM images of (**a**,**b**) TiO_2_ (10 nm)/Ag (10 nm). HRTEM images of TiO_2_ (t nm)/Ag (10 nm) bilayer, (**c**) t = 10 nm; (**d**) t = 20 nm; (**e**) t = 30 nm; (**f**) t = 40 nm.

The HRTEM images showed that TiO_2_ was amorphous and the spherical Ag particles with sizes between 5 nm and 10 nm were dispersed in the amorphous TiO_2_ matrix. Some Ag nanoparticles were partially or fully embedded in the TiO_2_ matrix and some remained on the surface, which led to the significantly rough surfaces (Figure 2b). When the TiO_2_ thickness increased, the ratio of Ag nanoparticles embedded in the TiO_2_ matrix increased and the size of the Ag nanoparticles also increased. When the Ag target was sputtered in the magnetic control system, it was difficult to form a continuous layer with an Ag thickness below 10 nm and many defects and holes formed in the film. Some high-energy Ag nanoparticles were able to penetrate into the TiO_2_ layer, forming a mixture structure with Ag nanoparticles fully-embedded or partially-embedded in the TiO_2_ matrix. When the TiO_2_ layer thickness further increased, the number of defects and holes decreased in the TiO_2_ layer and the diffusion barrier energy increased, which limited the diffusion of Ag and led to the growth of Ag particles.

**Figure 3:**
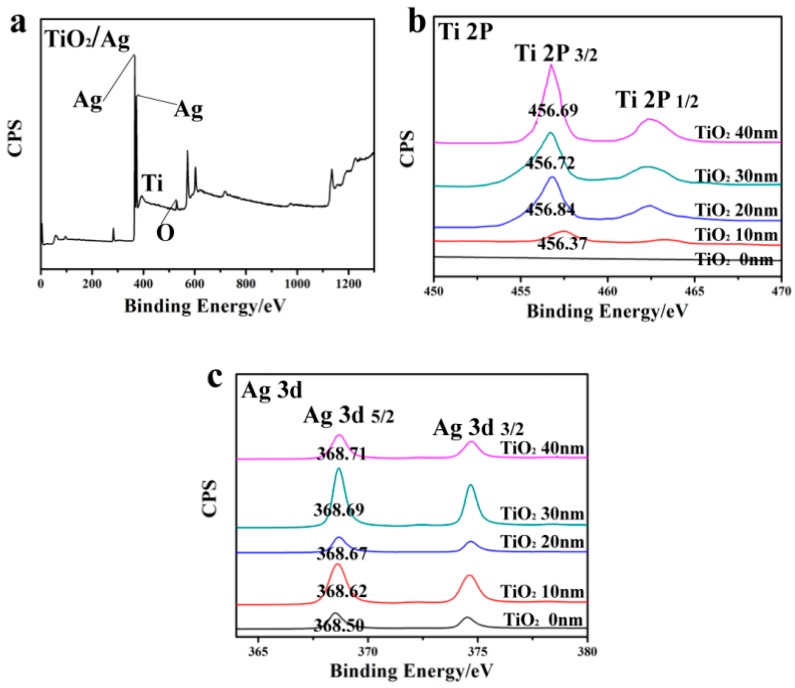
XPS images of (**a**) bilayer nanocaps TiO_2_/Ag; (**b**) Ti 2p and; (**c**) Ag 3d of TiO_2_(10–40 nm)/Ag (10 nm) on the polystyrene (PS) template for different thicknesses.

XPS measurements were carried out to identify the element composition of TiO_2_-Ag and analyze the chemical status of the elements. The survey XPS spectrum in Figure 3a shows the existence of Ti, Ag, and O elements in TiO_2_-Ag nanocaps, and the high-resolution XPS spectra of Ti 2p and Ag 3d are shown in Figure 3b,c. The Ti 2P spectrum consisted of two peaks identified as Ti 2P_3/2_ and Ti 2P_1/2_, respectively (Figure 3b). The binding energy of Ti moved in the direction of low binding energy when the TiO_2_ thickness increased, which suggested an increasing electron density for accepting some electrons. The peaks of Ag 3d_5/2_ and Ag 3d_3/2_ moved slightly towards the high binding energy (Figure 3c), which shows that the Ag lost some electrons and that the electron density decreased. The opposite shifts of binding energy for Ti 2p and Ag 3d indicated that some electrons transferred from metallic Ag to TiO_2_ owing to the interactions between the metal Ag and the semiconductor TiO_2_ [20]. However, in the Ag 3d spectra, the splitting of the Ag 3d binding energy was 6.0 eV, which indicated that the Ag mainly showed the Ag0 state in the TiO_2_-Ag nanocap structure, without the obvious oxidation of Ag. In this case, it is probable that the Ag nanoparticles part-embedded in the TiO_2_ matrix induced the formation of an Ag-O-Ti composite at the junction of TiO_2_ and Ag, which promoted the electron transfer from the surface of the Ag nanoparticle to the TiO_2_ [21].

**Figure 4:**
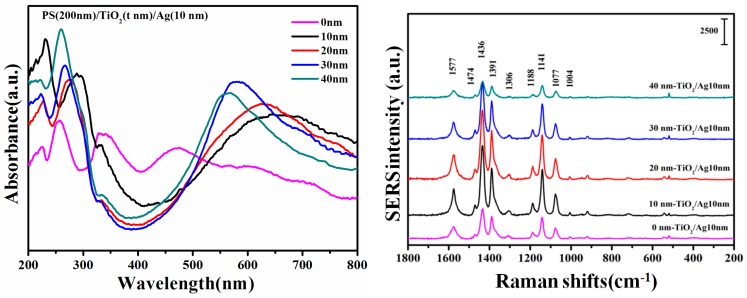
UV-Vis absorption and surface-enhanced Raman scattering (SERS) spectra of PS/TiO_2_ (0–40 nm)/Ag (10 nm) bilayer.

Compared to pure Ag, the TiO_2_-Ag nanocaps exhibited strong absorption in the UV and visible region, as shown in Figure 4. The absorption peaks of the Ag film at about 340 nm and 460 nm came from dipole resonance and quadrupole resonance, which broadened due to the coupling between the quadrupole resonance and the octupole resonance [22]. As the small Ag nanoparticles were almost trapped inside the TiO_2_ matrix and there was no exposure to oxygen, the excited electrons transferring from the surface of the Ag nanoparticle to the TiO_2_ conduction band remained in the Ag-TiO_2_ complex, leading to the increased concentration of free conduction electrons, which broadened the absorption band of the Ag-TiO_2_ complex. When the TiO_2_ thickness changed from 10 nm to 40 nm, the blue shifts of the resonance peaks were observed from 630 nm to 560 nm and became narrow. The absorption peak intensity first increased and then decreased, and the maximum was obtained when the TiO_2_ was 30 nm. With the increase of TiO_2_ thickness from 10 nm to 30 nm, more Ag nanoparticles were encaged in the TiO_2_ host matrix and began to agglomerate into large particles, which made the absorption peak narrow. When the TiO_2_ thickness increased to 40 nm, the absorption peak began to decrease, which may be attributed to the depressed plasmon absorption by TiO_2_. The observed blue shift was mainly because of the dielectric properties of the surrounding TiO_2_ and the interfacial electron transfer between Ag nanoparticles and TiO_2_. The Schottky barrier formed in the metal–semiconductor contact region due to the transfer of electrons from Ag to TiO_2_, which reduced the recombination rate of the electron-hole and improved the separation efficiency of the electron–hole pairs leading to the blue shift, consistent with the XPS results.

The SERS activities of the TiO_2_/Ag nanocap arrays were evaluated by the probe molecules 4-Aminothiophenol (PATP). The PATP molecules showed characteristic peaks located at 1004, 1077, 1141, 1188, 1306, 1391, 1436, 1474, and 1577 cm^−1^. Of these peaks, the peaks at 1077, 1188, and 1474 cm^−1^ were assigned to the ν(C-S), δ(C-H), and ν(C-C) stretching vibration, respectively, which were dominated by characteristic a1 vibrational modes [23,24,25]. The δ(C-H) at 1141 cm^−1^, [ν(C-C) + δ(C-H)] at 1391 and 1436 cm^−1^, and ν(C-C) at 1577 cm^−1^ were interpreted as b2 modes, as shown in Table 1 [26,27,28]. For pure Ag, the enhanced peaks were believed to be due to the roughness of the nanocap caused by Ag nanoparticles. However, decreases in the SERS intensity, for thicker TiO_2_ with 30 nm and 40 nm, were mainly caused by the reduced surface plasmon due to the embedment of the Ag nanoparticles in TiO_2_.

The SERS peak intensity first increased and then decreased when the TiO_2_ thickness changed from 0 to 40 nm. The charge-transfer (CT) process between the molecule and the TiO_2_/Ag substrate may play an important role in the change of SERS intensity. The degree of charge–transfer is used to evaluate the contribution of the chemical mechanism (CM) to the SERS intensity [29,30]. In the TiO_2_/Ag system, the peaks at 1077 cm^−1^ and 1436 cm^−1^ were chosen for CM analysis. The band at 1077 cm^−1^ is for the C-S stretching mode (a1 mode), which is totally symmetric to the SERS signal contributions. The other peak, 1436 cm^−1^ (b2 mode) is non-totally symmetric, because the adsorption effect and the SERS effect are affected by the CT process.

**Figure 5:**
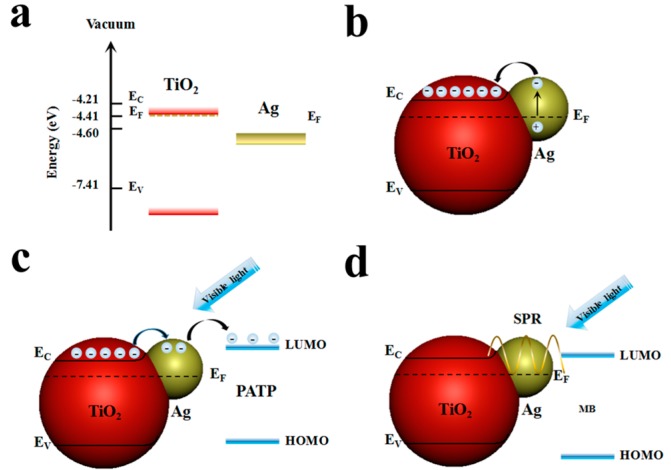
Schematic simulation mechanism of Ag/TiO_2_ for (**a**,**b**) the charge separation and transfer; (**c**) mechanisms of SERS; and (**d**) plasmonic photocatalysts.

According to the CT mechanism, non-vibration modes such as the b2 mode, are usually enhanced by the Herzberg–Teller contribution of CT, whereas the a1 model was not affected by the contribution of CT. In this case, changes in the CT process caused by various CM effects were qualitatively analyzed by PCT. The values of the degree of charge–transfer first increased from 0.693, to 0.746, 0.748, and then to 0.759, followed by a decrease to 0.751 when the TiO_2_ thickness was 0 nm, 10 nm, 20 nm, 30 nm, and 40 nm, which indicates the charge transition from the Fermi level of the TiO_2_/Ag composites to the lowest unoccupied molecular orbitals (LUMO) of the PATP molecules, as shown in Figure 5 [31].

## 4. Conclusions

In summary, a thin film of Ag was sputtered onto the TiO_2_ layer of different thicknesses to form the structure with Ag nanoparticles embedded in the TiO_2_ layer. The XPS peaks of Ti and Ag moved in opposite directions, indicating the changed electron density between the TiO_2_ and Ag, which in turn indicates the promotion of the electron transfer from the surface of the Ag nanoparticle to the TiO_2_ through the formation of the Ag-O-Ti composite. When TiO_2_ thickness changed from 10 nm to 40 nm, the UV spectra showed the blue shift resonance peaks from 630 nm to 560 nm and the maximum absorption peak intensity was obtained for the TiO_2_, namely 30 nm, due the controlled electron transfer process by the surrounding materials. The obvious SERS effects were observed, and the peak intensity first increased and then decreased when the TiO_2_ thickness changed, and the thickness-dependent changes were evaluated by the degree of charge–transfer. The observations of the XPS, UV absorption, and SERS effect were related closely to the dielectric properties of the metal-embedded structure and the interfacial electron transfer between the TiO_2_ semiconductor and Ag nanoparticles.

## Figures and Tables

**Table 1:** Wave numbers and assignment of bands in the SERS spectrum of the 4-Aminothiophenol (PATP) molecule.

Wavenumber (cm^−1^) PS/TiO_2_/Ag	Band Assignment
1577m	νCC, 8b(b_2_)
1474w	νCC, 19a(a_1_)
1436νs	νCC + δCH, 19b(b_2_)
1391s	δCH + νCC, 3(b_2_)
1306w	νCC + δCH, 14(b_2_)
1188w	δCH, 9a(a_1_)
1141νs	δCH, 9b(b_2_)
1077m	νCS, 7a(a_1_)
1004w	γCC + γCC, 18a(a_1_)

Approximate description of the modes (ν, stretch; δ and γ bend). Frequencies (in cm^−1^) followed by relative intensities (νs, very strong; strong; m, medium; w, weak).
